# Neuropeptide co-expression in hypothalamic kisspeptin neurons of laboratory animals and the human

**DOI:** 10.3389/fnins.2015.00029

**Published:** 2015-02-10

**Authors:** Katalin Skrapits, Beáta Á. Borsay, László Herczeg, Philippe Ciofi, Zsolt Liposits, Erik Hrabovszky

**Affiliations:** ^1^Laboratory of Endocrine Neurobiology, Institute of Experimental Medicine, Hungarian Academy of SciencesBudapest, Hungary; ^2^Department of Forensic Medicine, Clinical Center, University of DebrecenDebrecen, Hungary; ^3^Neurocentre Magendie, Institut National de la Santé et de la Recherche Médicale U862Bordeaux, France; ^4^Department of Neuroscience, Faculty of Information Technology and Bionics, Pázmány Péter Catholic UniversityBudapest, Hungary

**Keywords:** CART, dynorphin, hypothalamus, kisspeptin, neurokinin B, reproduction, substance P

## Abstract

Hypothalamic peptidergic neurons using kisspeptin (KP) and its co-transmitters for communication are critically involved in the regulation of mammalian reproduction and puberty. This article provides an overview of neuropeptides present in KP neurons, with a focus on the human species. Immunohistochemical studies reveal that large subsets of human KP neurons synthesize neurokinin B, as also shown in laboratory animals. In contrast, dynorphin described in KP neurons of rodents and sheep is found rarely in KP cells of human males and postmenopausal females. Similarly, galanin is detectable in mouse, but not human, KP cells, whereas substance P, cocaine- and amphetamine-regulated transcript and proenkephalin-derived opioids are expressed in varying subsets of KP neurons in humans, but not reported in ARC of other species. Human KP neurons do not contain neurotensin, cholecystokinin, proopiomelanocortin-derivatives, agouti-related protein, neuropeptide Y, somatostatin or tyrosine hydroxylase (dopamine). These data identify the possible co-transmitters of human KP cells. Neurochemical properties distinct from those of laboratory species indicate that humans use considerably different neurotransmitter mechanisms to regulate fertility.

## Reproductive significance of KP neurons in mammals

Hypothalamic neurons synthesizing kisspeptin (KP) play a pivotal role in the central regulation of puberty and reproduction. Inactivating mutations of the genes encoding for KP (*KISS1*) (Topaloglu et al., 2012) or its G-protein-coupled receptor (*KISS1R*; previously called GPR-54) (De Roux et al., 2003; Seminara et al., 2003) cause hypogonadotropic hypogonadism in humans. Impaired fertility has also been observed in *Kiss1* (D'anglemont De Tassigny et al., 2007; Lapatto et al., 2007)- and *Kiss1r* knock-out mice (Funes et al., 2003; Seminara et al., 2003) suggesting the highly conserved reproductive significance of KP/KISS1R-signaling in mammals. KP is a potent stimulator of adenohypophysial LH and FSH secretion (Navarro et al., 2005a,b). This action involves gonadotropin-releasing hormone (GnRH) and can be prevented with the GnRH receptor antagonist acyline (Gottsch et al., 2004; Shahab et al., 2005). The major effect of KP on GnRH-synthesizing neurons is direct. In various species, (i) KP-immunoreactive (IR) fibers establish appositions to GnRH neurons (Kinoshita et al., 2005; Clarkson and Herbison, 2006; Ramaswamy et al., 2008; Smith et al., 2008), (ii) GnRH cells express *Kiss1r* (Irwig et al., 2004; Han et al., 2005; Messager et al., 2005), (iii) GnRH neurons responds to KP with cFos expression (Irwig et al., 2004; Matsui et al., 2004) and depolarization (Han et al., 2005; Dumalska et al., 2008; Pielecka-Fortuna et al., 2008), (iv) the GnRH-specific *Kiss1r^−/−^* mice are infertile (Novaira et al., 2014) and (v) the infertile phenotype of global *Kiss1r^−/−^* mutant mice can be rescued via the selective reinsertion of *Kiss1r* into GnRH neurons (Kirilov et al., 2013).

## Functional subsets of KP neurons in laboratory species and the human

In a variety of mammals, two major populations of KP-synthesizing neurons exist in the anterior preoptic area and the arcuate nucleus (ARC), respectively (Lehman et al., 2010a). In rodents, the anterior preoptic cell group occurs as a periventricular continuum within the anterior periventricular (AVPV) and the periventricular preoptic (PVpo) nuclei (Clarkson and Herbison, 2006), together referred to as the KP neuron population of the rostral periventricular area of the third ventricle (RP3V) (Clarkson et al., 2008). Both KP cell populations are also detectable in the human, with the bulk of neurons in the infundibular (=arcuate) nucleus (Inf) (Rometo et al., 2007; Hrabovszky, 2013). KP neurons in the rodent RP3V and both KP cell groups in the human are sexually dimorphic, with higher cell number in females than in males (Clarkson and Herbison, 2006; Kauffman et al., 2007; Hrabovszky et al., 2010, 2011). Information accumulated in recent years indicate that both cell populations contain other neuropeptides and classic transmitters, in addition to KP. This review article discusses the available literature about these co-transmitters of KP neurons in laboratory species and humans, as also summarized in **Figure 2**.

## Co-transmitters and their receptors in KP neurons of laboratory species

### KP neurons of the anterior preoptic region

KP cells in the RP3V of female rodents have been implicated in positive estrogen feedback to GnRH neurons (Adachi et al., 2007; Herbison, 2008; Robertson et al., 2009). Immunohistochemical (IHC) and *in situ* hybridization (ISH) studies revealed that KP neurons in the RP3V express other neuropeptides as well as classic neurotransmitters.

#### Met-enkephalin (mENK)

Neurons IR to the proenkephalin (pENK)-derived opioid mENK overlap with KP-IR cells of the RP3V. Porteous et al. reported that, in adult female mice, dual-phenotype KP/mENK cells represent 28–38% of all KP-IR and 58–68% of all mENK-IR neurons in the AVPV and PVpo, respectively. These neurons give rise to dual-labeled axon varicosities which project to the preoptic area, the anterior hypothalamus and the ARC (Porteous et al., 2011).

#### Galanin

Recent ISH and IHC studies have established that galanin is also present in a subset of KP neurons in the RP3V of the mouse (Porteous et al., 2011; Kallo et al., 2012). In estrogen-treated ovariectomized mice, galanin was detected in 87%, and galanin mRNA in 38%, of KP neurons (Kallo et al., 2012). A lower incidence of colocalization was reported by Porteous et al.; in their study dual-labeled cells represented 7% of all KP-IR and 21% of all galanin-IR neurons both in the AVPV and PVpo of colchicine-pretreated female mice (Porteous et al., 2011).

#### Tyrosine hydroxylase (dopamine)

Tyrosine hydroxylase (TH) is a key and rate-limiting enzyme in catecholamine synthesis. Similarly to KP cells, TH-containing neurons in the RP3V are sexually differentiated in rodents (Kauffman et al., 2007; Clarkson and Herbison, 2011). In female rats, 20–50% of *Kiss1* mRNA-expressing cells in the RP3V, depending on the hormonal status, express *TH* mRNA (Kauffman et al., 2007). In female mice, 50% of KP cells contain TH immunoreactivity and vice versa, without noticeable variation between diestrus and proestrus. These KP/TH dual-phenotype neurons were also proposed to serve as the major source of dopamine in the synaptic regulation of GnRH neurons (Clarkson and Herbison, 2011).

#### GABA and glutamate

Dual-label ISH studies have established that about 20% of KP neurons in the mouse AVPV also express the mRNA encoding the glutamatergic marker type-2 vesicular glutamate transporter (vGluT2), whereas the mRNA of the GABA-synthesizing enzyme glutamic acid decarboxylase (GAD-67) was expressed in 75% of KP neurons (Cravo et al., 2011). These data indicate that AVPV KP neurons use amino acidergic, in addition to peptidergic and dopaminergic co-transmission.

### “KNDy” neurons in the ARC/Inf

Unlike the preoptic cell population, KP neurons of the ARC co-synthesize KP, neurokinin B (NKB) and dynorphin (Dyn) in several species (Burke et al., 2006; Foradori et al., 2006; Goodman et al., 2007; Navarro et al., 2011a; Bartzen-Sprauer et al., 2014), forming the basis for the “KNDy neuron” terminology (Lehman et al., 2010b). It is only becoming recognized lately that KNDy neurons do not consist of a homogenous cell population in that co-expression of the three KNDy peptides is only partial (Cheng et al., 2010; Hrabovszky et al., 2010, 2011, 2012; Overgaard et al., 2014). Morphological and electrophysiological studies provided evidence that KNDy neurons communicate extensively with each other (Burke et al., 2006; Foradori et al., 2006; Goodman et al., 2007; Navarro et al., 2011a,b; De Croft et al., 2013; Ruka et al., 2013). This local communication via NKB/neurokinin 3 receptor (NK3R) and Dyn/κ-opioid receptor (KOR) signaling was proposed to play a critical role in the generation of episodic GnRH/LH pulses (Navarro et al., 2009; Ohkura et al., 2009; Wakabayashi et al., 2010).

In addition to playing a putative role in the regulation of pulsatile GnRH/LH secretion, KNDy neurons have been implicated in negative sex steroid feedback action. Accordingly, neurotoxic ablation of KNDy neurons in rats prevented the rise in serum LH after ovariectomy (Mittelman-Smith et al., 2012). In some species like the sheep and primates, KNDy neurons might also be involved in positive estrogen feedback regulation. KP neurons in the ARC of ovariectomized ewes respond with cFos expression to estradiol treatment (Smith et al., 2009) and female rhesus monkeys with a disconnected mediobasal hypothalamus continue to respond to estrogen with LH and FSH surges (Krey et al., 1975; Plant et al., 1979). In contrast with the data from sheep, KP neurons in the preoptic area, but not the ARC, are activated during the positive estradiol feedback in goats (Matsuda et al., 2014).

Moreover, as reviewed by Rance et al., KP neurons in the ARC/Inf are also involved in the control of thermoregulation and their dysfunction is likely to contribute to the generation of hot flushes during menopause (Rance et al., 2013).

KP neurons of the ARC co-contain other neuropeptides, neuropeptide receptors and classic neurotransmitters in different laboratory species, as discussed below.

#### NKB and its receptor NK3R

The high reproductive significance of NKB, product of the human *TAC3* and mouse *Tac2* genes, has been recognized recently (Lasaga and Debeljuk, 2011). Mutations in the *TAC3* or *TACR3* genes encoding for NKB and its receptor NK3R, respectively, lead to hypogonadotropic hypogonadism and infertility in humans (Guran et al., 2009; Topaloglu et al., 2009). In laboratory species, large percentages of ARC/Inf KP neurons contain NKB or NK3R, although the reported colocalization patterns vary largely by species, sex and age (Burke et al., 2006; Goodman et al., 2007; Navarro et al., 2009, 2011a; Amstalden et al., 2010; Cheng et al., 2010; Ramaswamy et al., 2010; Wakabayashi et al., 2010; Hrabovszky et al., 2011, 2012; Overgaard et al., 2014). For example, while 90% of KP-synthesizing neurons in ovariectomized mice expressed *Tac2* mRNA signal and virtually all contained *Tac3r* signal, estradiol replacement decreased the incidence of NKB/Kiss1 co-labeled neurons to 53% and massively suppressed *Tac3r* mRNA expression (Navarro et al., 2009). In males, only half of the *Tac2*-expressing neurons expressed *Kiss1* mRNA, both in orchidectomized and testosterone-treated male mice (Navarro et al., 2011a). Similarly, a prominent group of NKB-only neurons was also detected in the caudal ARC in orchidectomized male, but not in ovariectomized female rats (Overgaard et al., 2014) and fibers single-labeled for NKB were also reported in the median eminence of female rats (True et al., 2011). The sex steroids estradiol (Navarro et al., 2009) and testosterone (Navarro et al., 2011a) regulate negatively *Tac2* and *Tac3r* expression in the ARC, which was proposed to decrease the activity of KNDy neurons by reducing a positive auto-feedback through NKB/NK3R signaling. Unlike in the above colocalization studies from rodents, only 40–60% of KP neurons co-expressed NKB immunoreactivity and NKB-only cells were not observed in the ARC of neonatally orchidectomized adult male monkeys (Ramaswamy et al., 2010).

While only NK1R and NK3R tachykinin receptors localized anatomically to KNDy neurons (Navarro et al., 2014), electrophysiological studies on male mice established that NKB stimulates the firing frequency of ARC KP neurons via activation of all three tachykinin receptor forms (NK1R, NK2R, NK3R) (De Croft et al., 2013). In contrast, in ovariectomized goats the NK3R receptor form plays the predominant role in the generation of GnRH pulses, with little, if any, contribution by NK1R and NK2R (Yamamura et al., 2015).

#### Dyn and its receptor KOR

The *Pdyn* gene product Dyn is the third KNDy peptide which was first colocalized with KP and NKB in the sheep; 95% of Dyn-IR cell bodies in ovariectomized and estrogen-treated ewes were also immunopositive for KP (Goodman et al., 2007). The extent of colocalization between KP and Dyn is also over 90% in the ARC of female mice, regardless of estrogen status (Navarro et al., 2009). ISH studies also revealed *KOR* mRNA, although only in relatively low subsets of KP neurons in female and male mice (Navarro et al., 2009).

#### Galanin

Galanin is co-expressed with KP not only in the RP3V (Porteous et al., 2011; Kallo et al., 2012) but also in the ARC (Kallo et al., 2012) of mice. In ovariectomized females, galanin mRNA was detected in 42.5%, and galanin immunoreactivity in 12.5%, of KP neurons (Kallo et al., 2012).

#### GABA and glutamate

ISH data indicate that KNDy neurons use amino acidergic co-transmitters and express *vGluT2* and *GAD*-*67* mRNAs (Cravo et al., 2011). Unlike KP cells of the RP3V which are mostly GABAergic (Cravo et al., 2011), the majority of KNDy neurons are glutamatergic (Cravo et al., 2011), in accordance with the IHC detection of vGluT2 in NKB/Dyn neurons of male and female rats (Ciofi et al., 2006).

## Immunohistochemical profiling of human KP neurons in the Inf

While neurochemical data about the preoptic KP cell population of the human are currently unavailable, a series of recent studies from our laboratory used immunofluorescent multiple-labeling to determine the phenotype of KP neurons in the Inf (Hrabovszky et al., 2010, 2011, 2012, 2013; Molnar et al., 2012; Skrapits et al., 2014). Colocalization experiments were carried out on autopsy samples from men of variable age groups as well as from postmenopausal women where expression is the highest for *KISS1* mRNA (Rometo et al., 2007) and KP immunoreactivity (Hrabovszky et al., 2010). Procedures of tissue processing, technical measures to maximize signals and avoid false-positive colocalization results, and details of the confocal analysis were described in the original publications. Here we extended the colocalization experiments to several other neuropeptides in order to obtain a fingerprint of KP co-transmitters/modulators. Samples were used from both men and postmenopausal women, in view that age and sex have strong effects on neuropeptide levels of KP neurons (Hrabovszky et al., 2011; Molnar et al., 2012). For reference to studies and primary antibodies, see **Figure 2**.

### Neuropeptides present in high percentages of human KP cells

#### Neurokinin B (NKB)

**Figure 1:**
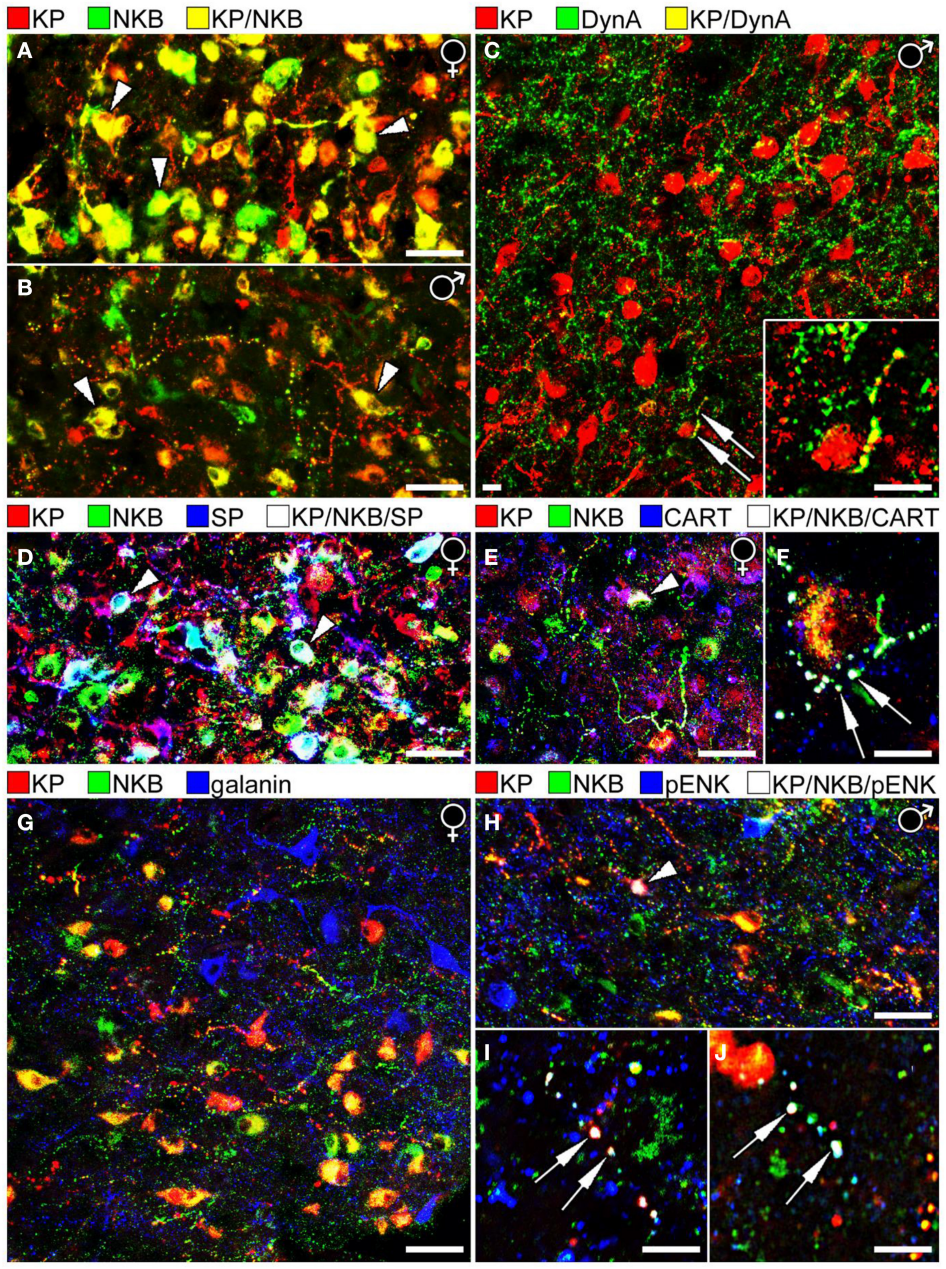
**Results of immunofluorescent studies to characterize the neuropeptide phenotype of human KP neurons in the Inf**. **(A,B)** The highest numbers of KP and NKB neurons and colocalization percentages can be observed in the Inf of postmenopausal women **(A)**. Compare to the weaker labeling of the Inf from a 67 year-old men in **(B)**. Note that the Inf contains many single-labeled axons (red and green), in addition to dual-labeled ones (yellow) in both sexes. **(C)** Unlike KP neurons of laboratory animals, the majority of human KP neurons do not contain Dyn A immunoreactivity. A rare case of dual-labeled KP/Dyn A axon (arrows) is shown at higher power in the inset. **(D)** KP neurons often contain SP immunoreactivity in postmenopausal women. White cells labeled by arrowheads correspond to KP/NKB/SP triple-phenotype neurons (Hrabovszky et al., 2013). **(E,F)** Similarly, CART is co-expressed in large subsets of KP and NKB neurons in postmenopausal women (Skrapits et al., 2014). Arrowhead in **(E)** and the two arrows in **(F)** point to a triple-labeled perikaryon and two axon varicosities, respectively. **(G)** The majority of neuropeptides tested in this study (see Figure 2), including galanin (blue), showed no colocalization with KP or NKB. **(H–J)** One exception was pENK which occurred in subsets of NKB-IR and KP-IR perikarya and fibers. Arrowhead in **(H)** points to a perikaryon, whereas arrows in **(I,J)** label axon varicosities that exhibit triple-neuropeptide phenotype (KP/NKB/pENK). The results shown in **(H,I)** vs. **(J)** were obtained with two different pENK antibodies (see text) to serve as positive control. (Note that the original color of the fluorochromes was changed so that KP is illustrated consistently in red). Scale bars: 40 μm in **(A,B,D,E,G,H)** and 12 μm in **(C,F,I,J)**.

Similarly to the ARC of laboratory animals, the Inf encloses a large population of KP neurons that also synthesizes NKB (Hrabovszky et al., 2010). The density of KP-IR (and NKB-IR) neurons in the Inf as well as the extent of their co-expression were found to be highly sex-dependent (Hrabovszky et al., 2011) (Figures 1A,B) and age-dependent (Molnar et al., 2012). As reviewed recently (Hrabovszky, 2013), the overall incidences of KP-IR and NKB-IR cell bodies are highest in postmenopausal women, lower in aged men and the lowest in young men. The percentage of KP perikarya containing NKB is similar in postmenopausal women (71%), aged men (>50 years; 78%) and young men (<50 years; 73%), whereas the percentages of NKB-IR perikarya with KP are highest in postmenopausal women (84%; Figure 1A), somewhat lower in aged men (68%; Figure 1B) and quite low in young men (36%), indicating that KP expression in NKB neurons is highly sex- and age-dependent. While KP expression might be suppressed in NKB neurons by testosterone in young men, it starts to increase with the decline of this negative feedback in aged individuals. The highest KP level and colocalization percentage are found in postmenopausal women where the inhibitory effect of estradiol is absent. Of note, considerable subsets of the KP-IR and NKB-IR fibers in all models are single-labeled (Hrabovszky et al., 2011; Molnar et al., 2012; Skrapits et al., 2014) and only 8–10% of KP-IR and NKB-IR axons forming appositions to GnRH neurons in young men and 25–30% in postmenopausal women contained both KP and NKB (Hrabovszky et al., 2011; Molnar et al., 2012).

**Figure 2:**
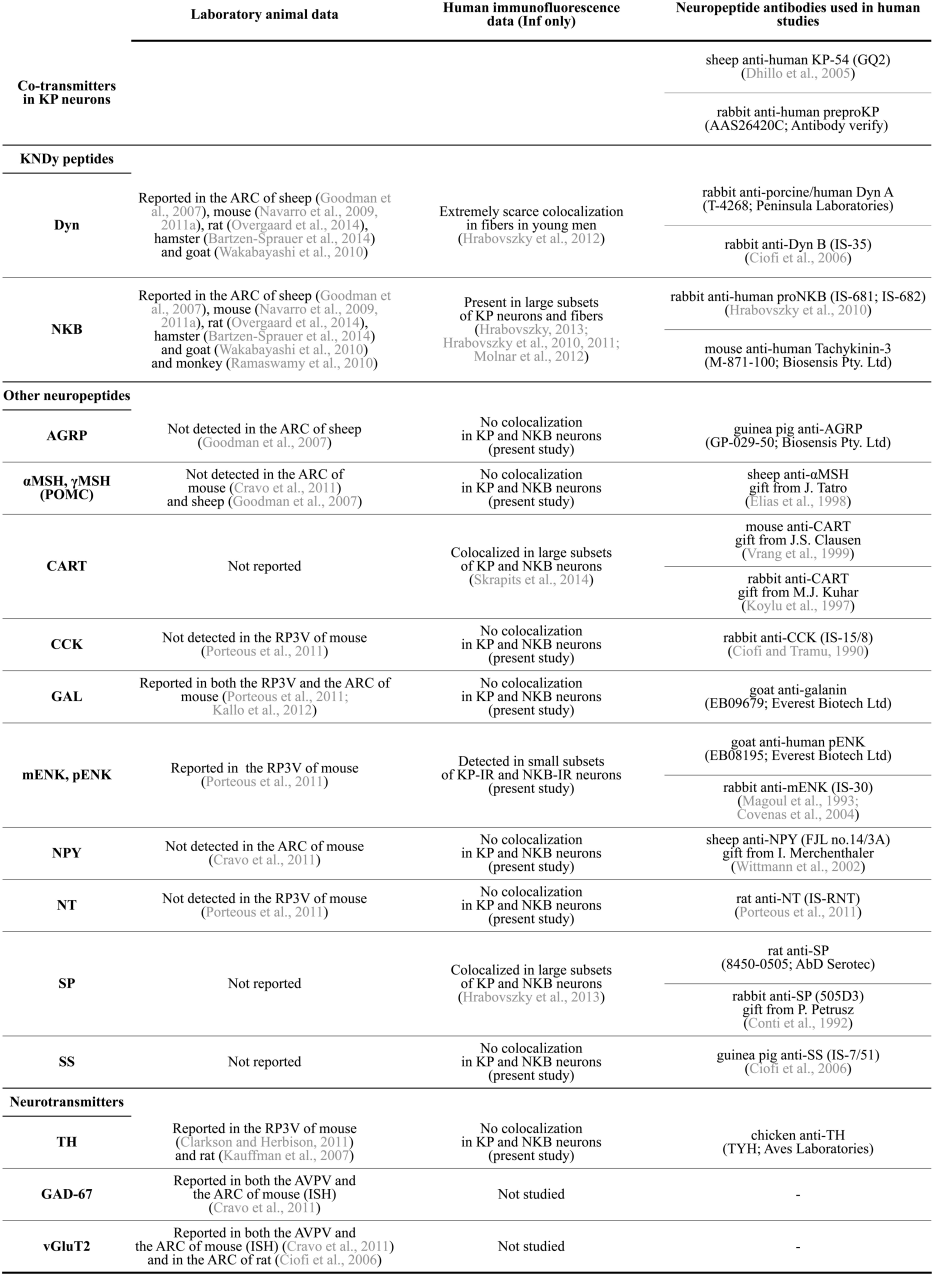
**Summary of neuropeptide/neurotransmitter co-expression data in KP neurons of laboratory animals and the human**. AGRP, agouti-related protein; αMSH, α-melanocyte stimulating hormone; ARC, arcuate nucleus; AVPV, anterior periventricular nucleus; CART, cocaine- and amphetamine regulated transcript; CCK, cholecystokinin; Dyn, dynorphin; GAD-67, glutamic acid decarboxylase-67; GAL, galanin; Inf, infundibular nucleus; ISH, *in situ* hybridization; KP, kisspeptin; mENK, met-enkephalin; NKB, neurokinin B; NPY, neuropeptide Y; NT, neurotensin; pENK, proenkephalin; POMC, proopiomelanocortin; RP3V, rostral periventricular area of the third ventricle; SP, substance P; SS, somatostatin; TH, tyrosine hydroxylase; vGluT2, type-2 vesicular glutamate transporter.

#### Substance P (SP)

The tachykinin peptide SP is derived from the *TAC1* gene and acts mainly via the NK1R. ISH studies by Rance et al. revealed that the *TAC1* and *TAC3* transcripts exhibit overlapping distribution in the Inf and both mRNAs increase remarkably after menopause (Rance and Young, 1991). Our laboratory used immunohistochemistry to analyze SP-IR neurons in the infundibular region (Hrabovszky et al., 2013). These studies demonstrated that the number and the staining intensity of SP-IR perikarya are significantly higher in postmenopausal women vs. age-matched men (Hrabovszky et al., 2013). We have shown a considerable overlap in the distribution of SP-IR, NKB-IR and KP-IR perikarya of postmenopausal women and we have established that SP immunoreactivity is present in large subsets of the KP-IR and NKB-IR neurons (Figure 1D); 31% of KP-IR and 25% of NKB-IR perikarya in this study contained SP, whereas 16.5% of all labeled cell bodies exhibited triple-neuropeptide phenotype. Dual- and triple-labeled fibers were also detectable in the infundibular stalk, raising the possibility of that these peptides are co-released into the portal circulation. Moreover, some of these axons established occasional contacts with hypophysiotropic GnRH-IR fibers in the postinfundibular eminence, the infundibular stalk and the neurohypophysis (Hrabovszky et al., 2013; Borsay et al., 2014). These anatomical observations suggest that SP may modulate the secretion of KP and NKB via autocrine/paracrine mechanisms and/or act on hypophysiotropic GnRH axons to regulate GnRH release directly.

#### Cocaine- and amphetamine-regulated transcript (CART)

CART has been implicated in the regulation of metabolic and neuroendocrine processes including reproduction (Rogge et al., 2008; Smith et al., 2010). In rodents, CART-IR fibers form close contacts with GnRH and KP neurons (Leslie et al., 2001; Rondini et al., 2004; True et al., 2013). Both KP and GnRH neurons respond to CART with depolarization. These data suggest that CART influences GnRH neuronal functions directly as well as indirectly, via modulating KP/KISS1R-signaling to GnRH cells (Roa and Herbison, 2012; True et al., 2013).

Colocalization studies of histological specimens from postmenopausal women provided evidence that KP-IR and NKB-IR perikarya often exhibit CART immunoreactivity (Skrapits et al., 2014); 48% of KP-IR and 30% of NKB-IR cell bodies were also CART-IR and 24% of labeled perikarya contained all three signals (Figure 1E). The colocalization phenomena were also detected at the level of fiber varicosities; 17% of KP-IR and 6% of NKB-IR fiber varicosities exhibited CART signal and 5% of all encountered varicosities were triple-labeled (Skrapits et al., 2014) (Figure 1F). Finally, some CART-containing KP and NKB fibers were also immunopositive for SP, indicating the overlap between the CART-IR and the SP-IR populations of KP and NKB neurons (Skrapits et al., 2014).

### Neuropeptides detected at low levels in human KP cells

#### Dynorphin (Dyn)

In ISH studies, *Dyn* expressing cells show a similar postmenopausal hypertrophy as do KP and NKB neurons in the Inf (Rometo and Rance, 2008). However, unlike in rodents and sheep where Dyn was detected in the vast majority of ARC KP cells, morphological studies from our laboratory only found poor evidence for the presence of Dyn immunoreactivity in KP neurons of young men (Hrabovszky et al., 2012) (Figure 1C). Here we have replicated these IHC colocalization experiments on histological samples obtained from postmenopausal women. These new studies were unable to reveal significant amounts of Dyn signal in KP-IR neurons or their processes, although scattered dual-labeled fibers were clearly detectable.

#### Proenkephalin (pENK)/met-enkephalin (mENK)

As part of an immunofluorescent screening for additional neuropeptides in human KP cells, we have addressed the putative colocalization between pENK and KP. To simultaneously visualize KP, NKB, and pENK with triple-labeling, a rabbit KP antiserum (Antibody Verify, Las Vegas, NV USA; AAS26420C; 1:1000), a mouse monoclonal NKB antibody (Biosensis Pty. Ltd, Thebarton, SA Australia; M-871-100; 1:3000) and an affinity-purified goat pENK antibody (Everest Biotech Ltd, Ramona CA USA; EB08195; 1:1000) were used. Tissue processing and IHC procedures were adapted from similar studies (Hrabovszky et al., 2010, 2011, 2012, 2013; Molnar et al., 2012; Skrapits et al., 2014).

While the confocal analysis provided no evidence for pENK expression in KP and NKB neurons of postmenopausal women (*n* = 3) being in line with a previous observation made in mice (Porteous et al., 2011), small subsets of KP-IR and NKB-IR neurons co-expressed pENK immunoreactivity (Figures 1H,I) in histological samples of human males (*n* = 3; age: 51, 64, and 78 years). The analysis of 212 KP and/or NKB perikarya established the presence of pENK signal in 12.5 ± 5.1% of NKB-IR and 1.9 ± 1.0% of KP-IR neurons. Co-expression was also studied in 700 axon varicosities with an approach adapted from our recent study (Skrapits et al., 2014). pENK signal was contained in 5.7 ± 2.5% of NKB-IR and 4.9 ± 1.8% of KP-IR axon varicosities. To confirm this colocalization phenomenon, the immunofluorescent detection of pENK in KP neurons has been replicated with a polyclonal rabbit mENK antiserum (IS-30; 1:1000) (Magoul et al., 1993), used in combination with a sheep polyclonal KP antibody (GQ2; 1:1000) (Dhillo et al., 2005) for dual-labeling (Figure 1J).

## Neuropeptides studied but not detected in human KP cells

The neurochemical phenotype of KP and NKB neurons was addressed in a large series of IHC experiments using antibodies against further neuropeptide targets. These colocalization studies failed to reveal galanin in human KP and NKB neurons (Figure 1G). This observation reveals a species difference from mice whose KNDy neurons express galanin mRNA and immunoreactivity (Kallo et al., 2012). In line with previous observations made in mice or sheep (Goodman et al., 2007; Cravo et al., 2011; Porteous et al., 2011), agouti-related protein, neuropeptide Y, α-melanocyte-stimulating hormone, neurotensin and cholecystokinin were not detectable in human KP and NKB neurons and these neurons were also immunonegative for somatostatin. Finally, human KP and NKB cells did not contain the dopaminergic marker tyrosine hydroxylase which was colocalized earlier with KP in the RP3V of rodents (Kauffman et al., 2007; Clarkson and Herbison, 2011).

## Conclusions

The functional significance of neuropeptide co-transmitters in KP neurons may vary largely. So far, NKB has been the most consistently detected co-transmitter of KP in the mediobasal hypothalamus, independently of species. Its critical involvement in the regulation of human reproduction is well established by the hypogonadotropic hypogonadism of the *TAC3*- or *TAC3R*-mutant humans (Topaloglu et al., 2009). In recent clinical studies, NKB/NK3R signaling was found essential during early sexual development and its importance became attenuated over time in sustaining the normal functioning of the reproductive axis (Gianetti et al., 2010). It is possible that compensatory mechanisms involve SP which is co-expressed in human KP cells (Hrabovszky et al., 2013) and other neurokinin receptors (NK1R, NK2R) that might substitute NK3R in human KP neurons. A second intensely studied neuropeptide, Dyn, is colocalized in the vast majority of KP neurons is rodents, sheep and goats, giving rise to the KNDy neuron terminology (Lehman et al., 2010b) and single-neuron models of the GnRH/LH pulse generator (Navarro et al., 2009; Ohkura et al., 2009; Wakabayashi et al., 2010). Somewhat surprisingly, our IHC studies did not reveal significant Dyn expression in KP neurons of young men (Hrabovszky et al., 2012) or postmenopausal women (present study). The low level of colocalization challenges the universal importance of endogenous Dyn in the regulation of episodic GnRH/LH secretion by KP neurons. The possibility exists that pENK/mENK we detected in human KP cells replaces some of the functions that Dyn plays in KP cells of laboratory species. The detection of SP, CART and pENK/mENK in KP neurons of the human which was not reported earlier in laboratory species indicates that humans and laboratory animals may use considerably different neuropeptide signaling mechanisms to regulate sex steroid feedback and the GnRH neurosecretory pulses. Finally, a large set of neuropeptides we have tested in this study for co-expression with KP do not seem to be present in KP neurons of any species studied so far. This peptide group includes neurotensin, cholecystokinin, proopiomelanocortin-derivatives, agouti-related protein, neuropeptide Y and somatostatin. Information about the neurochemical phenotype of human KP neurons summarized in this minireview will help us understand the peptidergic regulatory mechanisms of sex steroid feedback and episodic GnRH/LH secretion.

## Author contributions

Katalin Skrapits, Philippe Ciofi, Zsolt Liposits and Erik Hrabovszky conceived and designed the experiments and wrote the paper. Beáta Á. Borsay and László Herczeg contributed essential research material.

### Conflict of interest statement

The authors declare that the research was conducted in the absence of any commercial or financial relationships that could be construed as a potential conflict of interest.
